# Prevalence and Social Inequality in Youth Loneliness in the UK

**DOI:** 10.3390/ijerph181910420

**Published:** 2021-10-03

**Authors:** Pamela Qualter, Alexandra Hennessey, Keming Yang, Kayleigh L. Chester, Ellen Klemera, Fiona Brooks

**Affiliations:** 1 Manchester Institute of Education, University of Manchester, Oxford Road, Manchester M13 9PL, UK; alexandra.hennessey@manchester.ac.uk; 2 Department of Sociology, Durham University, Durham DH1 3LE, UK; keming.yang@durham.ac.uk; 3 Centre for Research in Public Health and Community Care, University of Hertfordshire, Hatfield AL10 9AB, UK; k.l.chester2@gmail.com (K.L.C.); fiona.brooks@uts.edu.au (F.B.); 4 Centre for Health Services Studies, Division of Law, Society and Social Justice, University of Kent, Canterbury CT2 7NZ, UK; E.Klemera@kent.ac.uk; 5 Faculty of Health, University of Technology Sydney, Sydney, NSW 2007, Australia

**Keywords:** loneliness, adolescents, youth, trends, prevalence, school, academic achievement, health

## Abstract

Using data from the English arm of the Health Behaviour in School-aged Children (HBSC) study, we examined the prevalence of loneliness for school-aged adolescents and how it is linked to social inequalities. The HBSC study collects data from 11-, 13-, and 15-year-olds, and is repeated every four years, allowing the exploration of prevalence rates of loneliness pre COVID-19 pandemic for comparison. We also explored whether loneliness was associated with socio-economic status (SES) and linked to academic attainment and health complaints. The total sample was 14,077 from 156 schools in England. Findings revealed a stable prevalence rate of 8.2% for loneliness from 2006 to 2014. We also found, across all survey years, (1) those aged 15 years were significantly lonelier than younger peers, (2) those who reported lower SES were lonelier than their more well-off peers, and (3) higher loneliness was associated with being ‘”below average” academically and reporting more health complaints. Conclusions: These prevalence data enable researchers, policymakers, and others to make comparisons with prevalence rates during the COVID-19 pandemic to explore whether there have been increases in loneliness among school-aged adolescents. Loneliness was consistently related to social inequalities, suggesting that targeted interventions that include whole systems changes are needed.

## 1. Introduction

There is concern world-wide that the COVID-19 pandemic and associated lockdowns, including school-closures, have increased loneliness among school-aged children and adolescents, and that will have increased the number of young people reporting mental health difficulties [1,2,3]. However, to be able to determine whether there has been an increase in loneliness during the COVID-19 pandemic and understand its impact, it is important to (1) have prevalence data before the pandemic for comparison, and (2) know whether loneliness is associated, not just with mental health, but with other important outcomes for youth, such as health and education, so that appropriate support are available for youth. Currently, in the UK there are no prevalence data on loneliness among school-aged adolescents of different ages, and no exploration of its relationship with health or educational outcomes. While there are data available for adolescents aged over 16 years, within the Community Life Survey for example [4] there is nothing comparable that provides prevalence data for adolescents still in school. In the current study, we fill that gap in the literature using the only pre-COVID-19 population data on loneliness among English school-aged adolescents.

Even before the COVID-19 pandemic, there were concerns about “an epidemic of youth loneliness” raised in the academic literature and popular press [5,6]. Those worries were fueled by increased awareness of evidence that the experience of loneliness is non-linear, following a U-shaped distribution across the lifespan, with those between 16 and 25 years and over 65 years reporting the highest loneliness [7]. Second, evidence from several large-scale surveys [4,8,9] supported that earlier work, showing that loneliness is highest among late adolescents and emerging adults (16–25 years). However, missing from the discussion was information on prevalence rates of loneliness during school-aged adolescents from population surveys. That gap in the literature has created a current issue given that teachers, policymakers, and charities want to know whether the prevalence of loneliness among adolescents has increased during the COVID-19 pandemic, and whether it is likely to have knock-on consequences for well-being and the support that needs to be put in place.

Within England, there are no national population surveys that include loneliness measures for school-aged adolescents with recent data available. However, there are survey data available from 2006–2014 that enable an examination of prevalence rates of loneliness for adolescents ages 11–15 years. In the current study, we analyse those data from the only English nationally representative survey that includes a measure of loneliness over time; the study includes 14,077 11-, 13-, and 15-year-olds from 156 schools in England. While we accept that the data are not recent (unfortunately, loneliness data were not collected in the HBSC in England in 2018), they are currently the only available data looking at loneliness among school-aged adolescents over time in England pre COVID-19. Thus, the use of these data is important, particularly given the current COVID-19 pandemic, where loneliness may have increased because of social isolation measures taken by the UK Government. Our study fills a knowledge gap by directly exploring the prevalence rate of loneliness among school-aged adolescents in England from 2006–2014. Such an exploration means that recent data exploring loneliness among youth during the COVID-19 pandemic can make a comparison with those earlier prevalence rates.

In addition to exploring the prevalence of loneliness for school-aged adolescents in England, we also explore whether loneliness is associated with academic achievement and health for those adolescents. There are empirical studies that have linked loneliness to poorer academic achievement [10] and poor physical health among youth [11]. However, those studies tend to look at older young people in university, have not explored the UK context, and/or do not use population-based data. In addition, only two studies with youth [12,13] have explored social conditions, specifically low socio-economic status (SES), and youth loneliness, finding that lower SES is associated with higher loneliness. Thus, the current study provides an important opportunity to address such gaps and explore the associations between loneliness among young adolescents and the following variables: academic achievement, health, and SES. Given that survey data were available for several years, with different cohorts, we were able to examine whether the associations between loneliness and SES, education, and health were robust.

### The Current Study

The current study had two aims. The first aim was to estimate the prevalence of loneliness reported by adolescents ages 11–15 years pre-COVID-19 pandemic. The second aim was to determine the relationships of health, education, and SES, with loneliness among that age group. Such exploration (1) provides information on rates of loneliness pre-COVID-19 from the only available dataset with adolescents ages 11–15 in England, and (2) helps establish whether loneliness is associated with health, education, and SES, in this age group, highlighting whether there is a need for intervention that promotes belongingness.

## 2. Materials and Methods

### 2.1. Sample

The HBSC study is repeated every four years (http://www.hbsc.org/about/index.html, accessed on 1 September 2021). The current study examines HBSC data for England for 2006, 2010 and 2014; the total sample was 14,522 11- to 15-year-olds from 156 schools (see Table 1 for sample breakdown). Loneliness data were available for 14,077 (97%) of the sample. Data on loneliness were not collected in the latest HBSC survey in 2018, making 2014 the most recent loneliness data we have available pre-COVID-19.

The sample frame for the HBSC study is young people attending school, aged 11, 13, 15 years. The sample in England was further stratified by region and school type, ensuring a large representative sample of young people from across independent and state schools. In 2014, there were 6181 eligible pupils registered in the participating classes; of those, 5679 returned at least a partially completed questionnaire resulting at a response rate of 92% at the pupil level (please see HBSC England National report 2014, www.hbscengland.org accessed on 1 September 2021); 5335 pupils from 50 schools had provided data on loneliness. In 2010, after data cleaning, a total of 4404 pupils, from 30 schools, remained in the survey, a response rate at the pupil level of over 90% was obtained. (please see HBSC England National report 2011, www.hbscengland.org accessed on 1 September 2021). In 2006, 4783 students from 76 schools in England took part in the survey; 4768 pupils remained in the current study, a response rate of 99.7%.

Overall sampling procedure and full details of sampling technique can be found in the HBSC International protocol [14]. Each country sample within the HBSC consists of approximately 1500 respondents in each age group. This is the minimum sample requirement according to the international HBSC research protocol to ensure a confidence interval of +/- 3% around a proportion of 50%, and taking account of the complex sampling design [15,16].

### 2.2. Measures

**Loneliness**. Loneliness was measured using the single item question “Thinking about the last week, have you felt lonely?” The response options were “never”, “seldom”, “quite often”, “very often” and “always”. The responses to the loneliness item can be collapsed to form a dichotomous category of “lonely” (‘very often” and “always”) and “non-lonely” (“never”, “seldom”, and “quite often”), enabling the differentiation between severe/prolonged loneliness from transient and no experiences of loneliness. The classification of transient versus prolonged loneliness has been shown to be useful for older adolescents [11] and adults [7], but different measures of loneliness and response options were used in those previous studies. Because we were conscious that the classification of responses into transient versus prolonged loneliness was subjective, we followed Rich-Masden et al., (2018) [13], recommendation to explore different cut-offs for determining lonely group membership, such that those who reported ‘quite often’ were included in the lonely group in our sensitivity analyses.

**Socio-Economic Status (SES).** The Family Affluence Scale (FAS) was devised as a proxy measure of family SES for use with adolescent samples. The FASII [12] consists of four items: “Does your family own a car, van or truck?” (no, yes—one, yes—two or more; scored 0–2), “Do you have your own bedroom?” (yes/no; scored 0–1), “During the past 12 months how many times did you travel away on holiday with your family?” (not at all, once, twice, more than twice; scored 0–3), and “How many computers does your family own?” (none, one, two, more than two; scored 0–3). Scores are summed to create a continuous composite scale, and are recoded to create a low (0–3), medium (4–6) and high (7–9) FAS group.

The FASII is reliable: young people accurately report on items in agreement with parents; it is sensitive, differentiating between SES groups; and has been validated across other SES measures such as parental occupation [17].

**Academic variables**. To capture academic and school engagement, three items were used: (1) academic achievement: “In your opinion, what does your class teacher(s) think about your school performance compared to your classmates?” (below average, average, good, very good; scored 1–4); (2) liking school: “How do you feel about school at present?” (I don’t like it at all, I don’t like it very much, I like it a bit, I like it a lot; scored 1–4); and (3) pressured by schoolwork: “How pressured (stressed) do you feel by the schoolwork you have to do?” (not at all, a little, some, a lot; scored 1–4).

**Health complaints**. The HBSC symptom checklist is an eight-item measure developed for use within the HBSC study [9], which captures a series of health symptoms: headache, stomach-ache, back-ache, feeling low, irritable or bad tempered, feeling nervous, difficulty in sleeping, and feeling dizzy. Respondents were asked if they had experienced any of these symptoms within the last six months and reported along a five-point scale (“hardly ever or never”, “about every month”, “about every week,” “more than once a week”, “about every day”). The scale demonstrates good test-retest reliability (r = 0.79) [18] and is often dichotomised into two or more health complaints more than once a week [19] capturing adolescents who are experiencing multiple health complaints.

### 2.3. Analyses Plan

First, prevalence rates for loneliness and family affluence were explored, i.e., year of dataset (2004, 2010, 2014), age, and gender. We used the Mantel-Haenszel linear-by-linear test, performed in SPSS, to assess whether prevalence trends were significant. We then ran a multi-level logistic regression, in Mplus 8.2, controlling for clustering at the school level (adolescents at the same school are more alike than adolescents at other schools), predicting loneliness from gender, age group, year of survey, and family affluence. Odds ratios (OR) for loneliness are reported. Analyses were initially run with groups of adolescents categorised as lonely (always and very often) and not lonely (quite often, seldom, and never). Interaction terms were included in the regression to determine whether any of the predictor variables differed by survey year, i.e., year*FAS, year*gender, year*age, and gender*age category. As recommended elsewhere [13], sensitivity analyses were run for all other cut-offs of loneliness to check the robustness of the findings.

We next examined whether loneliness, using the categories of ‘lonely’ (always and very often) and “not lonely” (quite often, seldom, and never), was associated with academic variables and health complaints. A series of multi-level multinomial regressions were conducted, with separate models for academic achievement, liking school, and feeling pressured by schoolwork. Gender, age group, year of dataset, and family affluence were all entered as covariates, and loneliness was the predictor in the analyse. Odds ratios for each academic outcome group are reported. For health, we explored whether having two or more health complaints a week was predicted by gender, age group, year of dataset, family affluence, and loneliness. Odds ratios (OR) for multiple health complaints are reported.

## 3. Results

Overall, 8.2% of children reported being lonely (categorised as ‘always’ and ‘very often’). Trends in loneliness over time were highest in 2006 (8.8%), reduced in 2010 (7.7%), but increased again in 2014 (8.0%). Thus, a linear-by-linear association was not observed between loneliness and year of dataset (Mantel–Haenszel linear-by-linear *X*^2^ = 1.79, *df* = 1, *p* = 0.182). However, those children who had “never” experienced feeling lonely in the last week reduced year by year (2006: 51.1%, 2010: 42.7%, 2014: 38.9%), and that trend was significant (Mantel–Haenszel linear-by-linear *X*^2^ = 145.60, *df* = 1, *p* < 0.001).

Multi-level logistic regression (Table 2) showed higher OR for loneliness among girls compared to boys (OR = 1.80, *p* < 0.001), higher OR for loneliness among 13-year-olds compared to 11-year-olds, and for 15 year olds compared to 13 year olds (OR = 1.22, *p* = 0.032 and OR = 1.76, *p* < 0.001, respectively). There was a small, but significant reduction in loneliness prevalence from 2006 to 2009–10 (OR = 0.79, *p* = 0.003). The interaction between survey year and gender was non-significant (OR = 1.15, *p* = 0.108), supporting our trend analysis that the relationship between loneliness and gender remained fairly constant over the survey years. The interaction between survey year and age was significant, showing that loneliness in 11-year-olds decreased across each survey year, reducing from 8% in 2006 to 4.4% in 2014 (OR = 1.26, *p* < 0.001).

### 3.1. Loneliness Is More Prevalent in Low Social Economic Groups

The prevalence of loneliness was associated with FAS group (Mantel-Haenszel linear-by-linear: *X*^2^ = 28.13, *df* = 1, *p* < 0.001). Across each survey year there was a higher proportion of children classified as lonely in the low FAS group compared to the high FAS group. Further exploration using multi-level multinomial logistic regression showed that children with medium, compared to high, FAS were more likely to be lonely (OR = 1.28, *p* = 0.002), and children categorised as low compared to high FAS 1.68 times more likely to be lonely (*p* = 0.001) [2].

### 3.2. Loneliness and School

Table 3 shows the results of the multi-level multinomial regression for predictors of the academic variables (achievement, liking school and pressured by schoolwork). Compared with their non-lonely peers, lonely adolescents were 3.11 times more likely to report being ‘below average’ academically (*p* < 0.001), 7.57 times more likely to report saying ‘I don’t like school at all’ (*p* < 0.001), nearly 4 times more likely to report ‘I don’t like school very much’ (OR = 3.90, *p* < 0.001), and nearly 4 times more likely to report feeling pressured by schoolwork ‘a lot’ compared to reporting ‘not at all’ (OR = 3.67, *p* < 0.001).

### 3.3. Loneliness and Health

Table 4 shows the predictors of two or more health complaints a week. Girls (OR = 1.64, *p* < 0.001), older adolescents (13-year-olds: OR = 1.32, *p* < 0.001; 15 year olds: OR = 1.65, *p* < 0.011), and those in the “medium” and “low” FAS groups (OR = 1.14, *p* = 0.002 and OR = 1.50, *p* < 0.001 respectively) experienced more health complaints than their peers. Adolescents classified as lonely were over 7 times more likely to experience two or more health complaints a week (OR = 7.47, *p* < 0.001).

## 4. Discussion

This examination of the HBSC data for England from 2006–2014 showed an average of 8% of adolescents aged 11–15 years reported loneliness “always” or “often”. In addition, we found the following: (1) loneliness was related to social inequality, suggesting that loneliness, consistent with previous work with adults [20] and adolescents [13], is, to some extent, about social comparison in terms of living conditions, at least in England; (2) that it was associated with lower than average performance at school, dislike of school, and feeling stressed at school, supporting recent work that showed schools are often difficult and lonely places for adolescents [21]; and (3) it was associated with self-reported poor health, suggesting that the association between loneliness and poor health starts early in life, and is not limited to older age. We also found that across all survey years, the oldest school-aged adolescents (those aged 15 years) were significantly lonelier than their peers. That finding supports previous work with non-population-based samples that suggests increasing loneliness during the adolescent years [22].

### 4.1. COVID-19 Context

Knowing the prevalence rate of loneliness among school-aged adolescents in England pre COVID-19 pandemic enables an accurate examination of whether loneliness has increased for that group during the COVID-19 pandemic. Indeed, early in the pandemic, Holmes et al., [18] noted that tracking loneliness and intervening early were important research priorities, and, while that analysis has been possible for older adolescents (16–24 years) and adults, where population surveys pre-COVID-19 included questions on loneliness, which has not been possible for school-aged adolescents in England. Our analyses of rates of loneliness for school aged adolescents pre-COVID-19 enables researchers, at least, to compare their COVID-19 loneliness rates for youth with those pre-COVID-19. Based on arguments put forward by Holmes et al. [23] and others [2], we could expect loneliness rates to have increased for youth during the pandemic, and for that to have impacted mental health. Our findings suggest that we might also expect to see consequences for educational outcomes and physical health too.

### 4.2. Limitations

We found that loneliness was associated with school outcomes and poor health. However, because HBSC collects data concurrently, while it is possible that loneliness impacts school outcomes, it is equally possible that under-achievement affects feelings of disconnection. Thus, prospective examination of the relationship between loneliness and academic outcomes is essential. That same argument can also be applied to the direction of effects between loneliness and health. This limitation also highlights the need for qualitative work that explores the experience of loneliness for school-aged adolescents: our quantitative approach limits what we can learn about social reality and causal links, and qualitative enquiry would enable more detailed exploration of the phenomenon.

How loneliness was measured in the HBSC study for England could be questioned. While recent research with adolescents has shown strong relationships between single-item, direct measures of loneliness and “the gold standard” measure of loneliness, the UCLA scale of loneliness [12], asking directly about loneliness is stigmatising, at least among adults, which could lead to under-reporting of loneliness [24].

We also acknowledge the fact that the available loneliness data only allowed examination of prevalence rates for loneliness among school-aged adolescents up until 2014. We do not know whether loneliness stayed stable from 2014 to March 2020 when lockdown measures were introduced in England. While there have been no large-scale country wide changes that likely affected the experiences of loneliness among school-aged adolescents in England from 2014 to 2020, it is possible that certain changes to the digital landscape and other contexts have precipitated changes. We acknowledge that loneliness data will be collected in the HBSC 2022 data collection, providing an opportunity to explore whether rates of loneliness among school-aged adolescents have changed since 2014. However, the impact of the COVID-19 pandemic even on self-reports in 2022 will need to be considered carefully.

### 4.3. Implications

Based on our findings, we recommend (a) the inclusion of loneliness item(s) in current population-based surveys with school-aged adolescents, so that current prevalence data can be calculated, (b) examination of prevalence rates of loneliness in different countries, exploring whether rates have changed over time, and (c) longitudinal studies to explore how loneliness, health, and academic outcomes are prospectively related. A recent meta-analysis highlighted the effects of interventions for lonely youth [25], and our findings support the notion that school is an ideal setting for those interventions given the association between loneliness and school outcomes.

## 5. Conclusions

We have provided pre-COVID-19 pandemic prevalence rates for loneliness for 11–15-year-olds in England so that comparisons with current rates during the COVID-19 pandemic can be made. We note that pre COVID-19, there was a consistently high prevalence of reported loneliness among school-aged adolescents, which is linked longitudinally to poor mental health [1] and we have shown that loneliness is linked to poorer health and education outcomes in our sample. Thus, even before COVID-19, there was a clear argument for investment in the design of suitable interventions for adolescents reporting loneliness.

## Figures and Tables

**Table 1:** The study population by survey year and gender, age group, prevalence of loneliness, and family affluence.

	Survey Year		
	2006	2010	2014
Pupils N	4783	4404	5335
Schools N	76	30	50
Gender %			
Boys	48.5	42.7	51.9
Girls	51.5	57.3	48.1
Age group %			
11-year-olds	34.7	33.8	39.8
13-year-olds	34.8	35.2	30.0
15-year-olds	30.5	31.0	30.2
Feel lonely ‘very often’ or ‘always’ %	8.8	7.7	8.0
Feels lonely ‘often’, ‘very often’ or ‘always’ % FAS group %	20.0	18.8	20.1
High	48.0	45.5	49.3
Medium	44.4	43.1	44.8
Low	7.6	11.3	6.0
Academic achievement %			
Below average	4.4	3.2	2.7
Average	23.6	23.5	21.9
Good	48.2	49.0	49.4
Very good	23.8	24.3	26.1
Liking school %			
I don’t like it at all	5.6	6.6	6.0
I don’t like it very much	12.0	15.1	13.5
I like it a bit	45.2	49.5	47.6
I like it a lot	37.1	28.7	32.9
Pressured by schoolwork %			
Not at all	10.9	15.2	15.4
A little	38.7	42.3	42.1
Some	27.8	25.7	26.1
A lot	22.6	16.8	16.5
Multiple Health Complaints% ^†^	30.4	32.7	29.0

Notes: ^†^ Two or more health complaints more than once a week. Across all survey years, those aged 15 years reported the highest rates of loneliness (mean sample reporting loneliness: age 11 years: 6.3%, age 13 years: 7.7%, and age 15 years: 10.9%). There was a significant linear-by-linear association between loneliness and age group (Mantel-Haenszel linear-by-linear *X*^2^ = 65.76, *df* = 1, *p* < 0.001), with that trend most evident in the 2014. Girls also reported higher rates of loneliness compared to boys (girls: 10.4%, boys: 5.7%; (*X*^2^ = 104.44, *df* = 1, *p* < 0.001, *Phi* = 0.09) being observed in 2006, 2010, and 2014.

**Table 2:** MLM logistic regression—Predictors of loneliness.

		Main Analyses	Sensitivity Analyses			Interaction Analyses
		OR (95% CI) for Lonely (Always, Very Often) Versus not Lonely (Quite Often, Seldom, Never)	OR (95% CI) for Lonely (Always) Versus not Lonely (Never, Seldom, Very Often, Quite Often)	OR (95% CI) for Lonely (Always, Very Often, Quite Often) Versus not Lonely (Never, Seldom)	OR (95% CI) for Lonely (Always, Very Often, Quite Often, Seldom) Versus not Lonely (Never)	OR (95% CI) for Lonely (Always, Very Often) Versus not Lonely (Quite Often, Seldom, Never)
Gender	If ‘girl’ compared to ‘boy’	**1.85 (1.64–2.087)**	**1.43 (1.16–1.76)**	**1.73 (1.60–1.87)**	**1.44 (1.35–1.54)**	1.39 (1.04–1.86)
Age Group	If ‘13 years’ compared to ‘11 years’	1.19 (1.03–1.37)	1.07 (0.83–1.38)	**1.19 (1.08–1.31)**	**1.20 (1.11–1.29)**	**0.76 (0.62–0.93)**
	If ‘15 years’ compared to ‘11 yrs’	**1.77 (1.54–2.02)**	**1.35 (1.05–1.72)**	**1.71 (1.56–1.88)**	**1.64 (1.52–1.77)**	**0.70 (0.50–0.98)**
Survey year	If ‘2010′ compared ‘2006′	**0.79 (0.67–0.92)**	**0.61 (0.46–0.81)**	**0.85 (0.75–0.96)**	**1.32 (1.18–1.48)**	**0.41 (0.29–0.59)**
	If ‘2014′ compared ‘2006′	0.95 (0.82–1.10)	0.89 (0.69–1.14)	1.06 (0.95–1.19)	**1.72 (1.55–1.91)**	**0.26 (0.13–0.49)**
FAS ^†^	If ‘medium’ compared ‘high’	**1.28 (1.14–1.43)**	**1.35 (1.08–1.67)**	**1.24 (1.15–1.35)**	**1.17 (1.10–1.25)**	**1.39 (1.10–1.76)**
	If ‘low’ compared to ‘high’	**1.68 (1.39–2.02)**	**2.66 (1.96–3.60)**	**1.61 (1.41–1.85)**	**1.31 (1.17–1.49)**	**1.99 (1.29–3.06)**
Interactions	Year*FAS					0.96 (0.87–1.06)
	Year*Gender					1.15 (1.01–1.32)
	Year*Age category					**1.27 (1.17–1.37)**

Notes: ^†^ FAS = Family Affluence Scale (FASII; Currie et al., 2008) [17], with scores summed to create the following groups: low family affluence (0–3), medium family affluence (4–6) and high family affluence (7–9); Estimates in bold are significant at *p* < 0.05.

**Table 3:** MLM multinomial regression—Predictors of School Variables.

Predictors of Academic Achievement				
		Academic Achievement (Reference Category ‘Very Good’) OR (95% CI)		
		If ‘below average’	If ‘average’	If ‘good’
**Gender**	If ‘girl’ compared to ‘boy’	**0.43 (0.36–0.52)**	**0.62 (0.56–0.68)**	**0.77 (0.72–0.83)**
**Age Group**	If ‘13 yrs’ compared to ‘11 years’	**2.35 (1.84–3.00)**	**1.43 (1.29–1.59)**	**1.16 (1.06–1.27)**
	If ‘15 yrs’ compared to ‘11 years’	**2.88 (2.27–2.67)**	**1.42 (1.27–1.58)**	1.03 (0.94–0.13)
**Survey year**	If ‘2010′ compared ‘2006′	**0.70 (0.53–0.92)**	0.94 (0.81–1.09)	0.99 (0.90–1.09)
	If ‘2014′ compared ‘2006′	**0.55 (0.42–0.71)**	0.80 (0.70–0.92)	0.92 (0.84–1.00)
**FAS** ^†^	If ‘medium’ compared ‘high’	**1.41 (1.17–1.70)**	**1.37 (1.25–1.50)**	**1.13 (1.05–1.22)**
	If ‘low’ compared to ‘high’	**1.81 (1.33–2.45)**	**1.37 (1.16–1.62)**	1.02 (0.88–1.17)
**Lonely**	If ‘lonely’ compared to ‘not lonely’	**3.11 (2.46–3.95)**	**1.37 (1.18–1.60)**	0.96 (0.83–0.10)
**Predictors of Liking School**				
		**Liking school (reference group ‘I like it a lot’)** **OR (95% CI)**		
		If ‘I don’t like it at all’	If ‘I don’t like it very much’	If ‘I like it a bit’
**Gender**	If ‘girl’ compared to ‘boy’	**0.70 (0.61–0.81)**	**0.82 (0.73–0.91)**	**0.87 (0.81–0.94)**
**Age Group**	If ‘13 yrs’ compared to ‘11 years’	**3.69 (3.04–4.47)**	**4.03 (3.53–4.60)**	**2.28 (2.10–2.48)**
	If ‘15 yrs’ compared to ‘11 years’	**7.09 (5.86–8.58)**	**6.38 (5.57–7.31)**	**3.23 (2.95–3.53)**
**Survey year**	If ‘2010′ compared ‘2006′	1.53 (1.14–2.06)	**1.64 (1.33–2.02)**	**1.47 (1.27–1.69)**
	If ‘2014′ compared ‘2006′	1.40 (1.07–1.83)	**1.47 (1.21–1.77)**	**1.32 (1.16–1.50)**
**FAS** ^†^	If ‘medium’ compared ‘high’	**1.33 (1.15–1.54)**	1.08 (0.97–1.20)	**1.13 (1.05–1.21)**
	If ‘low’ compared to ‘high’	**1.67 (1.30–2.16)**	**1.26 (1.04–1.53)**	**1.20 (1.05–1.39)**
**Lonely**	If ‘lonely’ compared to ‘not lonely’	**7.57 (6.19–9.24)**	**3.90 (3.26–4.65)**	**1.65 (1.41–1.93)**
**Predictors of Pressured by Schoolwork**				
		**Pressured by schoolwork (reference group ‘not at all’)** **OR (95% CI)**		
		If ‘a lot’	If ‘some’	If ‘a little’
**Gender**	If ‘girl’ compared to ‘boy’	**1.68 (1.50–1.88)**	**1.24 (1.12–1.38)**	**1.23 (0.17–1.42)**
**Age Group**	If ‘13 yrs’ compared to ‘11 years’	**1.83 (1.60–2.10)**	**1.85 (0.65–2.07)**	**1.33 (1.20–1.47)**
	If ‘15 yrs’ compared to ‘11 years’	**9.54 (8.19–11.11)**	**4.84 (4.21–5.57)**	**2.22 (1.94–2.54)**
**Survey year**	If ‘2010′ compared ‘2006′	**0.48 (0.40–0.57)**	**0.64 (0.55–0.74)**	**0.77 (0.68–0.87)**
	If ‘2014′ compared ‘2006′	**0.52 (0.43–0.61)**	**0.67 (0.59–0.77)**	**0.78 (0.69–0.88)**
**FAS** ^†^	If ‘medium’ compared ‘high’	**0.84 (0.74–0.94)**	0.93 (0.84–1.03)	0.96 (0.87–1.05)
	If ‘low’ compared to ‘high’	**0.64 (0.52–0.78)**	**0.72 (0.60–0.86)**	**0.64 (0.54–0.75)**
**Lonely**	If ‘lonely’ compared to ‘not lonely’	**3.67 (2.96–4.55)**	**1.56 (0.26–1.94)**	1.10 (0.89–1.36)

Notes: ^†^ FAS = Family Affluence Scale (FASII; Currie et al., 2004) [17], with scores summed to create the following groups: low family affluence (0–3), medium family affluence (4–6) and high family affluence (7–9); Estimates in bold are significant at *p* < 0.05.

**Table 4:** MLM logistic regression—Predictors of multiple health complaints.

		Main Analyses
		**OR (95% CI)** **for if ‘Experiencing more than Two Health Complaints more than Once a Week’**
**Gender**	If ‘girl’ compared to ‘boy’	**1.64 (1.53–1.76)**
**Age Group**	If ‘13 yrs’ compared to ‘11 years’	**1.32 (1.21–1.43)**
	If ‘15 yrs’ compared to ‘11 years’	**1.65 (1.51–1.79)**
**Survey year**	If ‘2010′ compared ‘2006′	1.10 (0.99–1.21)
	If ‘2014′ compared ‘2006′	0.94 (0.86–1.04)
**FAS** ^†^	If ‘medium’ compared ‘high’	**1.14 (1.06–1.23)**
	If ‘low’ compared to ‘high’	**1.53 (1.35–1.74)**
**Lonely**	If ‘lonely’ compared to ‘not lonely’	**7.47 (6.59–8.46)**

Notes: ^†^ FAS = Family Affluence Scale (FASII; Currie et al., 2004) [17], with scores summed to create the following groups: low family affluence (0–3), medium family affluence (4–6) and high family affluence (7–9); Estimates in bold are significant at *p* < 0.05.

## Data Availability

Funding contracts mean that data underlying this article are not publicly available. To gain access to the HBSC data, please contact The HBSC Data Management Centre (details provided at https://www.uib.no/en/hbscdata accessed on 1 September 2021).

## References

[1] Cooper K., Hards E., Moltrecht B., Reynolds S., Shum A., McElroy E., Loades M. (2021). Loneliness, social relationships, and mental health in adolescents during the COVID-19pandemic. J. Affect. Disord..

[2] Loades M.E., Chatburn E., Higson-Sweeney N., Reynolds S., Shafran R., Brigden A., Linney C., McManus M.N., Borwick C., Crawley E. (2020). Rapid Systematic Review: The Impact of Social Isolation and Loneliness on the Mental Health of Children and Ad-olescents in the Context of COVID-19. J. Am. Acad. Child Adolesc. Psychiatry.

[3] Scott S.R., Rivera K.M., Rushing E., Manczak E.M., Rozek C.S., Doom J.R. (2021). “I Hate This ”: A qualitative analysis of adolescents’self-reported challenges during the COVID-19 pandemic. J. Adolesc. Health.

[4] Office of National Statistics (2018). Loneliness—What Characteristics and Circumstances Are Associated with Feeling Lonely?. https://www.ons.gov.uk/peoplepopulationandcommunity/wellbeing/articles/lonelinesswhatcharacteristicsandcircumstancesareassociatedwithfeelinglonely/2018-04-10.

[5] Holt-Lunstad J. (2017). The Potential Public Health Relevance of Social Isolation and Loneliness: Prevalence, Epidemiology, and Risk Factors. Public Policy Aging Rep..

[6] Howe N. (2019). Millennials and the Loneliness Epidemic. Forbes.

[7] Victor C.R., Yang K. (2012). The Prevalence of Loneliness Among Adults: A Case Study of the United Kingdom. J. Psychol..

[8] Barreto M., Victor C., Hammond C., Eccles A., Richins M.T., Qualter P. (2021). Loneliness around the world: Age, gender, and cultural differences in loneliness. Pers. Individ. Differ..

[9] CIGNA (2019). New Cigna Study Reveals Loneliness at Epidemic Levels in America. https://www.cigna.com/about-us/newsroom/news-and-views/press-releases/2018/new-cigna-study-reveals-loneliness-at-epidemic-levels-in-america.

[10] Stoliker B., Lafreniere K. (2015). The Influence of Perceived Stress, Loneliness, and Learning Burnout on University Students’ Educational Experience. Coll. Stud. J..

[11] Eccles A.M., Qualter P., Madsen K.R., Holstein B. (2020). Loneliness in the lives of Danish adolescents: Associations with health and sleep. Scand. J. Public Health.

[12] Batsleer J., Duggan J. (2020). Young and Lonely: The Social Conditions of Loneliness.

[13] Madsen K.R., E Holstein B., Damsgaard M.T., Rayce S.B., Jespersen L.N., Due P. (2018). Trends in social inequality in loneliness among adolescents 1991–2014. J. Public Health.

[14] Currie C., Inchley J., Molcho M., Lenzi M., Veselska Z., Wild F. (2014). Health Behaviour in School-Aged Children (HBSC) Study Protocol: Background, Methodology and Mandatory Items for the 2013/14 Survey.

[15] Roberts C., Tynjälä J., Currie D., King M., Currie C., Samdal O., Boyce W., Smith R. (2004). Methods. People’s Health in Context: International Report from the HBSC 2001/2002 Survey.

[16] Roberts C., Currie C.E., Samdal O., Currie D.B., Smith R., Maes L. (2007). Measuring the health and health behaviours of adolescents through cross-national survey research: Recent developments in the Health Behaviour in School-aged Children (HBSC) study. J. Public Health.

[17] Currie C., Molcho M., Boyce W., Holstein B., Torsheim T., Richter M. (2008). Researching health inequalities in adolescents: The development of the Health Behaviour in School-Aged Children (HBSC) Family Affluence Scale. Soc. Sci. Med..

[18] Haugland S., Wold B. (2001). Subjective health complaints in adolescence—Reliability and validity of survey methods. J. Adolesc..

[19] Ottová-Jordan V., Smith O.R., Augustine L., Gobina I., Rathmann K., Torsheim T., Mazur J., Välimaa R., Cavallo F., Klanscek H.J. (2015). Trends in health complaints from 2002 to 2010 in 34 countries and their association with health behaviours and social context factors at individual and macro-level. Eur. J. Public Health.

[20] Szabo A., Allen J., Alpass F., Stephens C. (2019). Loneliness, socio-economic status and quality of life in old age: The moderating role of housing tenure. Ageing Soc..

[21] Verity L., Yang K., Nowland R., Shankar A., Turnball M., Qualter P. Loneliness from the Adolescent Perspective: A Qualitative Analysis of Conversations about Loneliness between Adolescents and Childline Counsellors. J. Adolesc Res..

[22] Qualter P., Vanhalst J., Harris R.N., van Roekel E., Lodder G., Bangee M., Maes M., Verhagen M. (2015). Loneliness Across the Life Span. Perspect. Psychol. Sci..

[23] Holmes E.A., O’Connor R.C., Perry V.H., Tracey I., Wessely S., Arseneault L., Ballard C., Christensen H., Silver R.C., Everall I. (2020). Multidisciplinary research priorities for the COVID-19 pandemic: A call for action for mental health science. Lancet Psychiatry.

[24] de Jong-Gierveld J., van Tilburg T., Dykstra P.A., Vangelisti A., Perlman D. (2006). Loneliness and social isolation. Cambridge Handbook of Personal Relationships.

[25] Eccles A.M., Qualter P. (2020). Review: Alleviating loneliness in young people—A meta-analysis of interventions. Child Adolesc. Ment. Health.

